# COVID-19 crisis in Cambodia: A dataset containing linked survey and administrative data of ninth-graders in rural areas

**DOI:** 10.1016/j.dib.2022.108476

**Published:** 2022-07-18

**Authors:** Esther Gehrke, Friederike Lenel, Claudia Schupp

**Affiliations:** a Agricultural Economics and Rural Policy, Wageningen University, Wageningen 6706KN, the Netherlands; b Chairgroup of International Economic Policy, University of Göttingen, Göttingen 37073, Germany; c Chairgroup for Behavioral and Experimental Economics, Ludwig-Maximilians-Universität München, München 80802, Germany

**Keywords:** COVID-19, Schooling, Cambodia

## Abstract

Between 2019 and 2021, we collected detailed administrative data of grade 9 students from 54 lower-secondary schools in rural Cambodia. We also collected phone-survey data from these students in July and August 2020, to understand the implications of the nation-wide lockdown, that was implemented to curb the spread of COVID-19 in March 2020. The administrative data contain information on students’ grades, school characteristics and teacher characteristics from the school year 2019–2020, as well as information about the students’ enrollment in high school after the end of the school year (January 2021). This information is available for 3258 students. The phone-survey data contains information on students’ socio-economic background, parental education and occupation, as well as students’ study behavior, time use, educational aspirations and expectations, and perceptions of the COVID-19 crisis. This information is available for 2197 students.

## Specifications Table

**Table:** 

Subject	Microeconomics, Economics of Education
Specific subject area	Students’ study behavior, time use and perceptions of COVID-19, and their relationship with parental background, experience of economic hardships in the family, and academic performance.
Type of data	Stata data file (.dta)
	Comma delimited file (.csv)
How data were acquired	Phone-survey and administrative data, both collected by research assistants
	Instruments: Open Data Kit (survey) and Excel (administrative data)
	Questionnaire available in the supplementary material
Data format	Raw data
	Coded data
Parameters for data collection	Administrative data were collected from grade 9 students of 54 lower-secondary schools across four provinces in Northwest Cambodia. Sample schools were eligible for high school scholarships provided by Child’s Dream, an international NGO (65% of schools), or similar in characteristics to these schools and from neighboring districts (35% of schools). For the phone survey, one or two classes of grade 9 were randomly selected to participate in the phone-survey, of these classes all students were contacted. Students were asked for informed consent before the interview.
Description of data collection	Administrative data were collected on a monthly basis by a total of three local research assistants, who contacted school principals and class teachers during a period of 18 months. Phone-survey data were collected between July and August 2020. Eligible students were called by local interviewers (under the supervision of two team leaders, one supervisor, and the principal investigators).
Data source location	Four provinces in Northwest Cambodia: Banteay Meanchey, Battambang, Oddar Meanchey, and Siem Reap
Data accessibility	Repository name: OpenICPSR
	Data identification number: 10.3886/E170281V1
	Direct URL to data: https://www.openicpsr.org/openicpsr/project/170281/version/V1/view
Related research article	E. Gehrke, F. Lenel, C. Schupp, COVID-19 Crisis, Economic Hardships and Schooling Outcomes, Educ. Financ. Policy. (2022) 1–51. https://doi.org/10.1162/edfp_a_00378.

## Value of the Data

• The main strength of this dataset is its combination of phone-survey data collected during the nation-wide COVID-19 lockdown (July-August 2020) with detailed administrative data collected before and after the phone survey. This dataset contains comprehensive information about students’ socio-economic characteristics, parental occupation before and during the COVID-19 pandemic, as well as students perceptions of the pandemic.
• Researchers, practitioners, and education officials can make meaningful use of the data to understand the implications of the COVID-19 pandemic on students from low-income settings, and to link experiences made during the crisis and perceptions of the crisis to academic performance before and after the pandemic.
• Since the dataset is linked at the individual level with administrative information on academic performance and school, teacher, and class characteristics, it can also be used to study shock experiences at the group versus individual level.

## 1 Data Description

The data contain information about students of grade 9 from rural Northwest Cambodia that was collected between 2019 and 2021. The data collection was designed to understand the implications of the nation-wide lockdown—implemented in March 2020 to curb the spread of COVID-19—on students school performance, as well as on educational and career aspirations. The first source of data is administrative data that cover the entire (prolonged) school year (November 2019 to November 2020) and the transition to high school in January 2021. The second source is data from a phone survey that was conducted during July and August 2020 with a subset of these students.

The dataset is available in STATA format and in open-source format (CSV), and is complemented by the code to replicate [1] (STATA do-file), as well as the phone-survey questionnaire.

### 1.1 Administrative data

The variables in the administrative data are summarized in Table 1, Table 2, Table 3. This dataset contains information on students’ gender, age, and whether she or he is a class leader (maximum of three per class) as summarized in Table 1. In addition, it contains monthly information on grades in the subjects Khmer, Math, English, and the total over all subjects, as well as days absent, covering the time-period from November 2019 until March 2020. The maximum points for Khmer, Math, and English are 100, 100, and 50, respectively. The total grade was standardized within a class by the authors, as the number of subjects included in that total grade varies across schools.

**Table 1:** Student characteristics and grades.

Variable	Mean	Median	SD	Min	Max	Obs.
Student Characteristics						
Female student (adm. data)	0.53	1.00	0.50	0.00	1.00	3258
Age (adm. data)	15.06	15.00	1.32	11.00	20.00	3258
Student is class leader	0.02	0.00	0.15	0.00	1.00	3258

Grades and Absences before Lockdown						
Khmer grade in November	60.59	63.00	22.22	0.00	100.00	1278
Math grade in November	53.28	53.00	27.39	0.00	100.00	1207
English grade in November	22.60	24.00	16.00	0.00	50.00	1176
Total grade in November	389.31	396.00	101.96	0.41	683.00	1278
Absences in November	0.96	0.00	1.60	0.00	11.00	2224
Khmer grade in December	58.06	58.00	21.80	0.00	100.00	3245
Math grade in December	46.92	47.00	26.53	0.00	100.00	3245
English grade in December	17.22	15.00	14.40	0.00	50.00	3169
Total grade in December	343.84	328.00	117.36	7.50	771.00	3245
Absences in December	1.45	1.00	2.30	0.00	15.00	2892
Khmer grade in January	55.99	58.00	23.02	0.00	100.00	3178
Math grade in January	47.37	49.00	25.77	0.00	100.00	3138
English grade in January	17.52	18.00	13.89	0.00	50.00	3127
Total grade in January	347.31	337.00	120.01	20.00	767.00	3238
Absences in January	1.60	1.00	2.42	0.00	17.00	2930
Khmer grade in February	55.82	55.00	22.02	0.00	100.00	3034
Math grade in February	50.26	50.00	24.47	0.00	100.00	3034
English grade in February	16.06	15.00	13.74	0.00	50.00	2978
Total grade in February	343.44	333.00	115.94	4.00	762.00	3096
Absences in February	1.73	1.00	2.70	0.00	19.00	2874
Khmer grade in Semester 1	57.66	58.00	19.07	0.00	99.00	1928
Math grade in Semester 1	52.88	51.33	23.02	0.00	100.00	1928
English grade in Semester 1	16.96	15.00	12.44	0.00	50.00	1774
Total grade in Semester 1	286.26	281.50	73.38	41.00	786.33	1928

Constructed Variables						
Semester 1 exam was conducted before school closure	0.45	0.00	0.50	0.00	1.00	3202
Total grade in November (std. within class)	0.03	0.06	0.97	-5.33	2.63	1278
Total grade in December (std. within class)	0.04	0.02	0.95	-4.32	3.26	3245
Total grade in January (std. within class)	0.02	-0.00	0.97	-3.60	3.49	3238
Total grade in February (std. within class)	0.00	0.02	0.99	-4.66	3.10	3096
Total grade in Semester 1 (std. within class)	0.02	-0.05	0.96	-3.14	5.53	1928

Grades and Dropout after Lockdown						
Participated in final exam	0.88	1.00	0.33	0.00	1.00	3258
Khmer grade in final exam	65.77	66.00	16.37	5.00	100.00	2864
Math grade in final exam	56.13	55.00	21.20	4.00	100.00	2864
English grade in final exam	6.73	4.00	7.86	0.00	50.00	2792
Total grade in final exam	325.01	315.00	64.79	118.00	533.00	2864
Ranks in top 20% in final exam	0.17	0.00	0.38	0.00	1.00	3258
Ranks in top 15% in final exam	0.13	0.00	0.34	0.00	1.00	3258
Ranks in top 10% in final exam	0.09	0.00	0.28	0.00	1.00	3258
Ranks in top 5% in final exam	0.04	0.00	0.20	0.00	1.00	3258
Dropped out during school closure	0.12	0.00	0.33	0.00	1.00	3258
Student passed final exam (cond. on part.)	0.95	1.00	0.22	0.00	1.00	2864
Transitioned to high school	0.83	1.00	0.38	0.00	1.00	3163

**Table 2:** Distances.

Variable	Mean	Median	SD	Min	Max	Obs.
No. of students living in 1 km radius	13.44	11.00	10.21	1.00	44.00	3258
Distance school to Province town	42.53	30.96	29.35	6.26	102.86	3258
Distance school to District town	10.81	7.59	9.36	0.07	41.16	3258
Distance school to border	36.06	19.40	36.39	0.26	102.29	3258
Dist. village to district town	11.74	9.28	9.09	0.11	48.38	3258
Dist. village to province town	42.63	31.37	29.36	0.62	111.78	3258
Dist. village to school	3.47	2.16	3.73	0.00	24.53	3258

**Table 3:** School and teacher characteristics.

Variable	Mean	Median	SD	Min	Max	Obs.
School Characteristics						
No. of grade 9 classes	1.96	2.00	0.86	1.00	4.00	3258
No. of students per class	49.29	50.00	11.62	23.00	78.00	3258
School partners with Child’s Dream	0.62	1.00	0.49	0.00	1.00	3258
Enrollment of grade 9 in 2018	90.83	77.00	48.43	22.00	205.00	3258
Enrollment of grade 9 in 2019	95.17	78.00	50.17	31.00	202.00	3258
Dropout rate grade 9 in 2018	0.13	0.13	0.08	0.00	0.34	3258
Dropout rate in grade 9 2019	0.17	0.17	0.07	0.00	0.31	3258
Transition rate to high school in 2017	75.02	80.65	17.86	25.53	100.00	2368
Transition rate to high school in 2018	76.50	80.00	15.96	20.34	100.00	2940

Teacher Characteristics						
Female Teacher	0.30	0.00	0.46	0.00	1.00	3258
Age of teacher	32.26	31.00	6.06	20.00	57.00	3258
Teacher’s yrs. of experience at resp. school	9.21	8.50	5.88	1.00	33.00	3258
Teacher has univ. degree	0.49	0.00	0.50	0.00	1.00	3258
Distance betw. school and teacher’s home	7.81	3.86	9.12	0.16	41.09	3258
Distance between teacher’s and student’s home	8.91	6.09	8.90	0.00	48.31	3258

Flood						
No. of days floodwater within 30 km of school (10 weeks)	37.50	39.00	9.46	14.00	50.00	3258
No. of days floodwater within 30 km of school (school-return week)	4.10	4.00	1.57	1.00	7.00	3258
No. of days floodwater within 10 km of school (10 weeks)	15.55	13.00	11.36	2.00	39.00	3258
No. of days floodwater within 10 km of school (school-return week)	1.46	1.00	1.32	0.00	5.00	3258
No. of days floodwater within 5 km of school (10 weeks)	8.89	4.00	9.17	0.00	31.00	3258
No. of days floodwater within 5 km of school (school-return week)	0.44	0.00	0.87	0.00	4.00	3258

In addition to monthly grades, this dataset contains students’ grades in the first midterm exam (Semester 1 exam) and in the lower-secondary graduation exam (final exam). The midterm exams usually determine whether the student will be allowed to participate in the final exam. This requirement was relaxed in 2020 due to the COVID-19 crisis. In some schools, the first midterm exam was conducted just before the lockdown (as indicated by the variable “Semester 1 exam was conducted before school closure”), in others this exam could not be conducted anymore. In that case, the Semester 1 grade is the average of the months December, January, and February.^1^ The final exam is a standardized, national exam consisting of 11 subjects. The total grade of the final exam is not standardized by the researchers as it’s content is comparable across schools. Students pass the exam with 260 points and above. We also created dummy variables that indicate whether a particular student participated in the final exam (2864 students did so), whether the student ranked among the top 20%, 15%, 10%, or 5% of students in the final exam (the means are somewhat lower that the respective percentiles because this variable is coded as zero for students who did not participate in the final exam), whether the student passed the final exam (i.e. obtained more than 260 points), and finally whether the student dropped-out during school closure, which is defined as one if the student did not participate in the final exam. The variable indicating whether the student transitioned to high school has fewer observations than the overall sample, as this information was collected from lower-secondary teachers (and in some cases students’ classmates) after the new school year started in 2021. Not in all cases could teachers or classmates say with certainty if a particular student had indeed enrolled in high school.

The administrative data also include information on the location of the students’ homes (village name). We used this information to calculate the number of students living within a radius of 1 km of each other, as well as the geodesic distance of the students’ hometown to the school, to the district capital, to the province capital, and to the (Thai) border. For privacy reasons, we do not disclose the location of the students’ village or of the school, but only the distances between those, see Table 2.

In terms of school and class characteristics, the dataset contains the class size (as of February 2020), information on the number of classes in grade 9 at the lower-secondary school, the dropout rate in grade 9 in previous years, and the share of students of grade 9 that transitioned to high school in previous years (all obtained from the school’s principal),^2^ the class teacher’s age, gender, and experience at this particular school, whether the class teacher has a university degree, and the distance at which the teacher lives from the school (all collected from the class teacher). The variables are summarized in Table 3.

Finally, this dataset contains the number of flood days experienced at the school during the first week that students were allowed to return to school after the first lockdown (September 7, to 13, 2020) as well as during the entire period between school reopening and completion of the final exam (10 weeks), as a severe flood hit the region right at this time (also summarized in Table 3). To construct these variables, we collected daily flood maps from MODIS Near Real-Time Global Flood Mapping Project [2], and constructed two sets of variables: the first-school-week variables range from 0 to 7, and indicate the number of days in that first school week on which a flooded area was detected within a 5, 10, or 30 km radius around the school. The variables for the total period range from 0 to 70, and indicate the total number of days in that entire 10-week period on which a flooded area was detected within a 5, 10, and 30 km radius around the school.

### 1.2 Phone-survey data

**Fig. 1:**
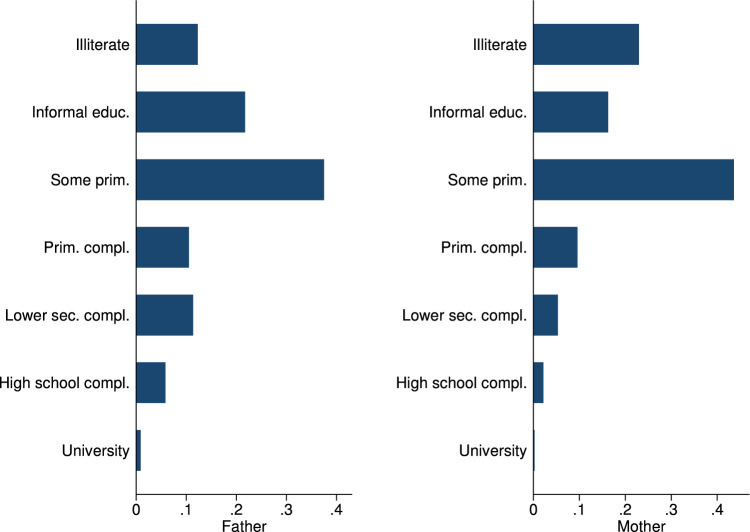
Parental education.

**Fig. 2:**
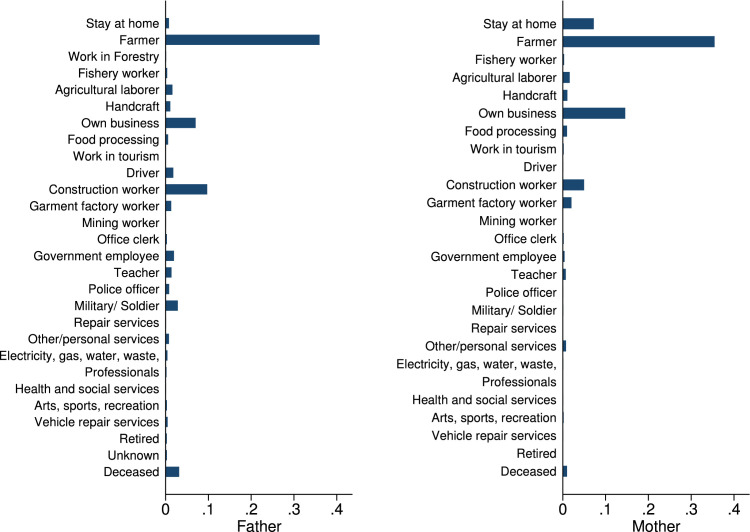
Parental occupation.

**Fig. 3:**
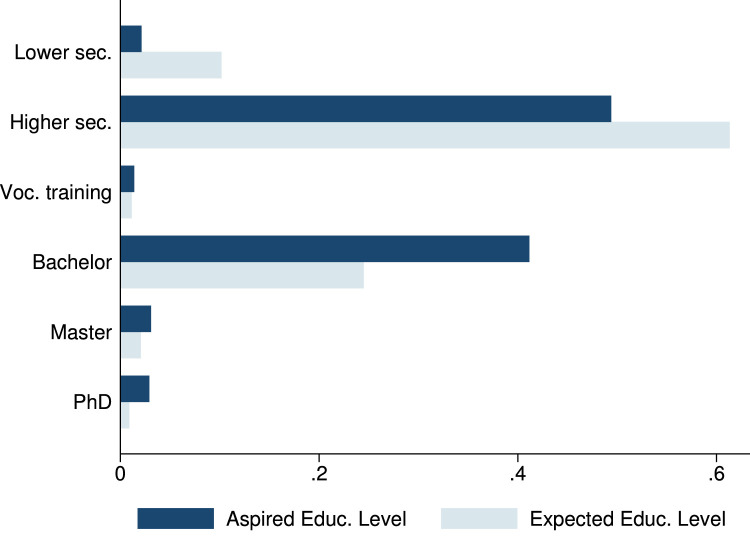
Students’ educational aspirations and expectations.

**Fig. 4:**
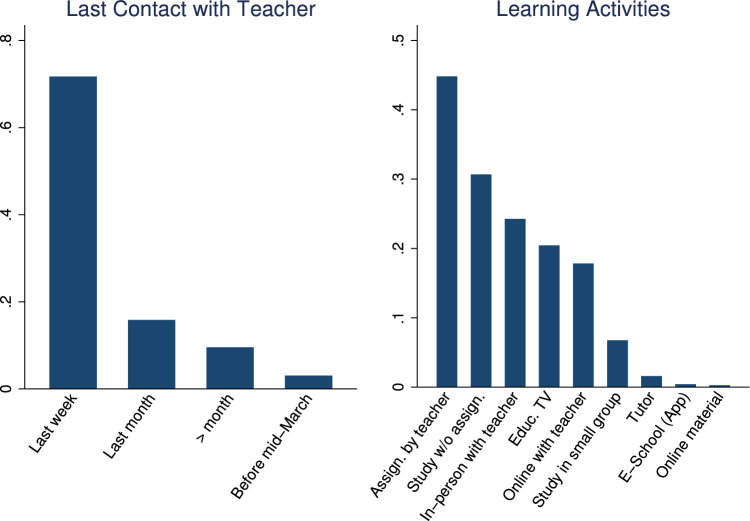
Students’ study behavior during lockdown.

**Fig. 5:**
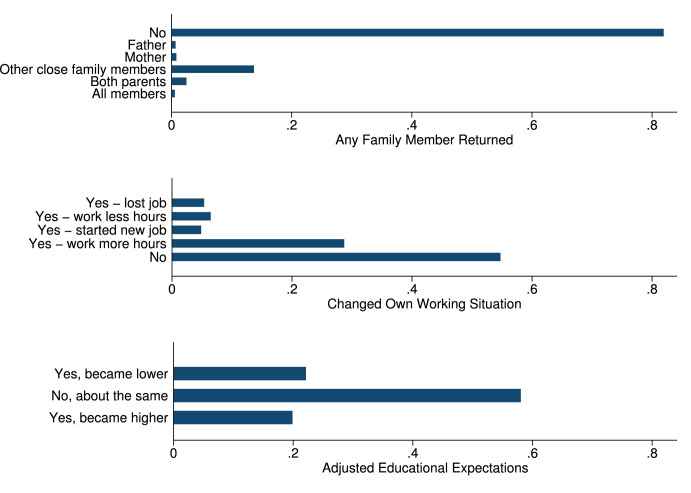
Impact of COVID-19 on students.

**Fig. 6:**
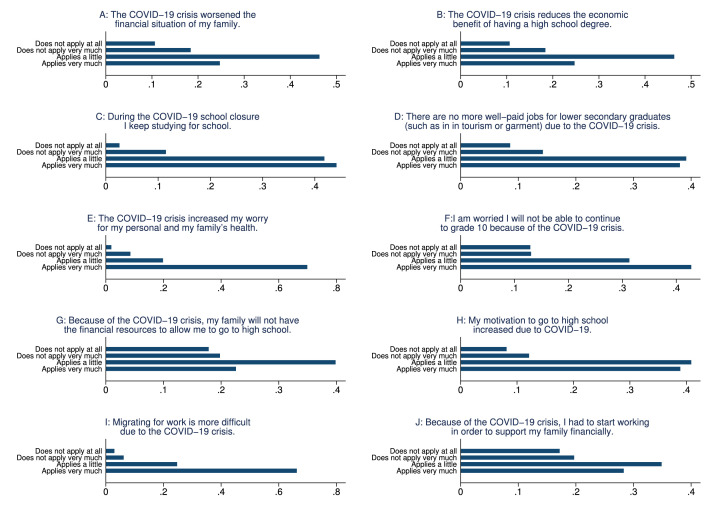
Students’ perceptions of COVID-19.

In the phone survey, we collected information on student and family characteristics, on students’ study behavior, time-use, educational aspirations and expectations, as well as COVID-19 perceptions. These variables are summarized in Table 4, as well as in Fig. 1, Fig. 2, Fig. 3, Fig. 4, Fig. 5, Fig. 6.

**Table 4:** Phone-survey data.

Variable	Mean	Median	SD	Min	Max	Obs.
Student Characteristics						
Age (survey data)	15.66	16.00	1.15	12.00	23.00	2197
Gender (survey data)	0.55	1.00	0.50	0.00	1.00	2197
Smartphone ownership	0.85	1.00	0.36	0.00	1.00	2197
Any family member migrated	0.36	0.00	0.48	0.00	1.00	2196

Aspirations and Expectations						
Highest educational level wanted (in schooling years)	13.53	12.00	2.06	9.00	20.00	2177
Likelihood of achieving educational level (0–10)	5.82	5.00	1.55	0.00	10.00	2175
Expected educational level in schooling years	12.52	12.00	2.03	8.00	20.00	2174
Likelihood of getting preferred job (0–10)	5.57	5.00	1.53	0.00	10.00	2140

Cost Expectations						
Costs of high school (1000 Riel)	290.95	260.00	181.50	10.00	4000.00	1960
Most expensive item in high school:						
*Transportation*	0.09	0.00	0.29	0.00	1.00	2135
*Accommodation and food*	0.27	0.00	0.44	0.00	1.00	2135
*Extra classes*	0.59	1.00	0.49	0.00	1.00	2135
*School material*	0.05	0.00	0.22	0.00	1.00	2135
Costs for most expensive item (1000 Riel)	128.72	100.00	124.76	6.00	3000.00	1997
Application for scholarship:						
*Yes*	0.21	0.00	0.41	0.00	1.00	2161
*No, no scholarship was available this year*	0.17	0.00	0.37	0.00	1.00	2161
*No, did not know of any scholarships*	0.18	0.00	0.38	0.00	1.00	2161
*Don’t know what a scholarship is*	0.04	0.00	0.20	0.00	1.00	2161
*No, for other reasons*	0.40	0.00	0.49	0.00	1.00	2161

Activities during Lockdown						
Returning to school after lockdown:						
*Yes*	0.96	1.00	0.20	0.00	1.00	2195
*Maybe*	0.03	0.00	0.16	0.00	1.00	2195
*No*	0.01	0.00	0.11	0.00	1.00	2195
Studied last 7 days	0.90	1.00	0.30	0.00	1.00	2165
Worked last 7 days	0.57	1.00	0.50	0.00	1.00	2195
Hrs. worked last 7 days	5.60	6.00	2.36	1.00	14.00	1245
Main activity in last 7 days: study	0.24	0.00	0.43	0.00	1.00	2193
Main activity in last 7 days: paid work	0.11	0.00	0.31	0.00	1.00	2193
Main activity in last 7 days: hh work	0.62	1.00	0.48	0.00	1.00	2193
Main activity in last 7 days: leisure	0.02	0.00	0.15	0.00	1.00	2193

COVID and Parental Working Situation						
Heard about COVID-19	0.99	1.00	0.08	0.00	1.00	2196
Father experienced income loss (=1)	0.63	1.00	0.48	0.00	1.00	2194
Mother experienced income loss (=1)	0.65	1.00	0.48	0.00	1.00	2194
Father changed job(s)	0.11	0.00	0.31	0.00	1.00	2192
Mother changed job(s)	0.09	0.00	0.29	0.00	1.00	2192
Father exp. income loss (probability)	0.65	0.63	0.10	0.44	0.95	2033
Father exp. income loss (prob. of main occ.)	0.66	0.63	0.10	0.44	0.95	2033
Father exp. income loss (av. probability)	0.66	0.63	0.10	0.44	0.95	2033
Father exp. income loss (WB probability)	0.80	0.81	0.09	0.55	1.00	2016
Mother exp. income loss (probability)	0.68	0.63	0.08	0.44	0.95	1918
Mother exp. income loss (prob. of main occ.)	0.68	0.63	0.08	0.44	0.95	1918
Mother exp. income loss (av. probability)	0.68	0.63	0.07	0.44	0.95	1918
Mother exp. income loss (WB probability)	0.82	0.81	0.04	0.55	1.00	1903

Perceptions of COVID-19 Crisis						
Index for financial worries related perceptions	2.75	2.67	0.70	1.00	4.00	2193
Index for studying related perceptions	2.78	2.67	0.56	1.00	4.00	2159
Index for job prospects related perceptions	2.80	3.00	0.47	1.00	4.00	2175

Participation in Lottery						
Participation in lottery	0.94	1.00	0.24	0.00	1.00	2195
Choice of prize (1=Educational mentoring)	0.82	1.00	0.38	0.00	1.00	2064

In terms of student and family characteristics, we collected the age and gender of the student and whether she or he has a smartphone, as well as the migration incidence in the family (see Table 4). We also collected information about parental education and parental occupation, as summarized in Figs. 1 and 2.

Further, we inquired about students’ aspirations and expectations regarding the highest level of schooling, and what kind of job students would like to do as adults (open ended). For educational aspirations and expectations, the dataset contains the original variables (with categorical answers, see Fig. 3) as well as constructed variables that translate the categories into years of schooling (see Table 4). The likelihood variables allow students to state their confidence in the achieving their aspired educational level or career on a scale from 0 (not at all confident) to 10 (extremely confident).

We also collected students’ expectations about the costs of high school, what students expect the most expensive item to be, the cost of that item, and whether they had already applied for a high school scholarship (see Table 4). We then asked about students’ motivation to return to school after the lockdown. Information about students’ study behavior during the lockdown includes regularity of contact with the teacher, and in what type of remote learning activities the student was involved in (see Fig. 4). Time-use information contains whether the student studied in the last 7 days, whether the student worked in the last 7 days (and if yes, how many hours), and what the student’s main activity was in the last 7 days (see Table 4).

In terms of COVID-19 effects, we collected information about whether one (which one) or both parents lost their job due to COVID-19, or whether one had lower income (see Table 4). In case one or both parents lost their job, we also asked about the pre-crisis occupation of the parent. These questions are similar to what has been collected in other COVID-19 related phone surveys [3]. We use this information in three ways: first, from the survey answers, we construct the pre-crisis occupation of each parent (initial job) according to the ISIC Rev.4 classification [4]. Second, from the income/job loss questions, we calculate dummy variables (mother/father experienced income losses/changed job(s)) that equal one if the respective applies (see Table 4. Third, we calculate the probability of experiencing income losses per sector of occupation, and assign this value to each parent based on her/his initial occupation as described in Gehrke et al. [1].

The data also contain information about whether a migrating family member was forced to return to Cambodia, whether students had to change their working situation, and whether students adjusted their educational expectations due to COVID-19 (see Fig. 5).

In addition, the data include information about students’ perceptions of the crisis evaluated on a 4-point Likert scale, as summarized in Fig. 6. We constructed three indices from these statements by averaging over the level of agreement to the respective sub-questions. The financial worries index is the average agreement to the statements A, G, and J. The job prospects index is the average of the statements B (reversed), D, and I. The studying index is the average of the statements C, F (reversed), and H.

Finally, the data contain information entered by the interviewer concerning the quality of the call, whether the student was motivated during the call, and whether she or he seemed to have understood the questions.

### 1.3 Other variables

A few additional variables are included in the data for replication purposes (see Table 5). The variable priority class equals one if a student’s class was (randomly) selected to receive the intervention described in Gehrke et al. [5], or was selected to serve as control class.^3^ Student participated in RCT equals one if the student was present on the day of the intervention. Unique combination of paternal/maternal occupations are categorical variables used to cluster standard errors in Gehrke et al. [1].

**Table 5:** Other variables.

Variable	Mean	Median	SD	Min	Max	Obs.
Priority classes	0.49	0.00	0.50	0.00	1.00	3258
Student participated in RCT	0.24	0.00	0.43	0.00	1.00	3258
Student participated in phone survey	0.67	1.00	0.47	0.00	1.00	3258

## 2 Experimental Design, Materials and Methods

### 2.1 Sample

The sample consists of students of grade 9 from rural lower-secondary schools in Northwest Cambodia. Students of grade 9 are usually about 15 years old, and in their final compulsory schooling year. To select the schools we collaborated with Child’s Dream, an NGO that offers high-school scholarships in the study area. We received the contact information from the schools that Child’s Dream was working with (*i.e.* schools whose students were eligible for their scholarships), and of those schools sampled all schools with more than 30 students in grade 9 (resulting in a sample of 39 schools across 4 provinces: Banteay Meanchey, Battambang, Oddar Meanchey, and Siem Reap). To increase sample size, we added 21 schools from other districts, but in the same provinces, to our sample. These schools have similar characteristics to the schools that partner with Child’s Dream. We were able to receive students’ administrative records and school characteristics from 54 of the 60 sampled schools; in six schools the principals were not willing or able to share this information. Within the sample schools, we then aimed at collecting information from all grade 9 students in at least one class. Some schools have more than one class, in those we randomly selected one (in some cases two) classes to be part of our sample.

This sample of students is not representative of students at lower-secondary schools in rural Cambodia. However, at least in terms of school characteristics, our data are broadly comparable to the average school in rural areas of the country, as discussed in Gehrke et al. [1].

### 2.2 Timeline

Prior to our study, we contacted the principals of all schools and asked for permission to conduct a study at their school. If willing to participate, we collected a few school characteristics from the principal, as well as the contact details of the class teacher of the selected class.

The administrative data were collected from the class teacher on a monthly basis since November 2019. Around this time, we randomly assigned half of the schools to be part of an educational intervention that was conducted between February and March 2020 [5].

On March 16, 2020 a national lockdown was announced to slow the spread of the COVID-19 virus. At that time, we had successfully visited and conducted a half-day workshop at 18 schools. Schools were closed until further notice, and the government quickly set up a remote learning system which encouraged students to keep studying during the school closure via TV programs and teacher assignments.

A few months into the first lockdown, we conducted a phone survey with students in our sample. Our target sample were all students from the administrative sample that had still been participating in exams just before the first lockdown (n=3258). Participation in the phone survey was entirely voluntary, and we explained the aim of the project and asked students for consent to participate in the study before starting the interview. We were able to reach 2197 students (response rate = 67%).

Shortly after the phone survey was completed, schools were reopened for grade 9 students for a period of two months (between September 2020 and November 2020) to allow students to prepare for their final exam, which determines admission to high school. Students had about 8 weeks to prepare for the final exam, which took place in November 2020 and was spread over a period of two weeks. A few weeks after the final exam was completed, the Prime Minister announced that all students who had participated in the final exam were allowed to transition to high school irrespective of whether they had obtained more than 260 points [6]. The new school year started in January 2021; however schools were closed again after a few weeks as a new lockdown was imposed.

Between September 2020 and July 2021, we continued to collect administrative data to learn about the student’s performance in the final exam, and—after the new school year started—whether students had enrolled in high school. The process of data collection is also detailed in Gehrke et al. [7].

## Ethics Statement

This data collection obtained ethical approval from the Ethics Committee of the University of Göttingen (IRB approval date 11 February 2020), as well as from the Social Sciences Ethics Committee at Wageningen University and Research (IRB approval date 25 May 2020). Participants gave consent to the interview over the phone.

## CRediT authorship contribution statement

**Esther Gehrke:** Conceptualization, Methodology, Validation, Investigation, Formal analysis, Writing – original draft. **Friederike Lenel:** Conceptualization, Methodology, Validation, Investigation, Writing – review & editing. **Claudia Schupp:** Conceptualization, Methodology, Investigation, Formal analysis, Data curation, Writing – review & editing.

## Declaration of Competing Interest

The authors declare that they have no known competing financial interests or personal relationships which have, or could be perceived to have, influenced the work reported in this article.

## Data Availability

COVID-19 crisis in Cambodia: A dataset containing linked survey and administrative data of ninth-graders in rural areas (Original data) (Mendeley Data).

## Appendix A Supplementary materials

Supplementary Data S1 Supplementary Raw Research Data. This is open data under the CC BY license http://creativecommons.org/licenses/by/4.0/ Supplementary Data S1

## Footnotes

1 The Semester 2 exam was waived for all students.

2 Note that not all principals could provide all information on previous years.

3 We randomly assigned 30 schools to treatment and 30 to control. In each school, one class was then selected randomly to be treated (in treatment schools) or to serve as control class (in control schools). In some schools one priority class and one additional class are included in the data (for the purpose of evaluating spillover effects). While the additional classes are part of the phone survey, they were not prioritized by the interviewers (and response rates are relatively low in these classes).
