# Supplementary material for: COVID-19 crisis in Cambodia: A dataset containing linked survey and administrative data of ninth-graders in rural areas
Source: Data Brief. 2022 Jul 18;43:108476. doi: 10.1016/j.dib.2022.108476 (PMC9309421; doi:10.1016/j.dib.2022.108476)
Supplement: Supplementary Data S1 — Supplementary Raw Research Data. This is open data under the CC BY license http://creativecommons.org/licenses/by/4.0/ [file mmc1.pdf]

Questionnaire: COVID-19 crisis in Cambodia

type	name	label::English (en)	label::Khmer (kh)
integer	fu_interviewerid	Interviewer ID	អត្តលេខអ្នកសម្ភាសន៍
integer	fu_schoolid	School ID	អត្តលេខសាលារៀន
integer	fu_studentid	Student ID	អត្តលេខសិស្ស
note	fu_note_family	Start with Interview Before we get started, I will collect some basic information about you:	 សូមចាប់ផ្តើមការសម្ភាសន៍ មុនពេលយើងចាប់ផ្តើម ខ្ញុំសូមសួរសំណួរខ្លះអំពីប្អូន ៖
integer	fu_age	1) How old are you?	1) តើប្អូនអាយុប៉ុន្មានឆ្នាំ?
select_one sex_list	fu_sex	2) What is your gender?	2) តើប្អូនភេទអ្វី?
select one yes_list	fu_smartph	3) Do you own a smart phone?	3) តើប្អូនមានទូរស័ព្ទស្មាតហ្វូនប្រើដែរឬទេ?
select_one educ_list	fu_fa_att	4) What is the highest educational degree of your father?	4) តើឪពុករបស់ប្អូនរៀនបានថ្នាក់ទីប៉ុន្មាន?
select_multiple occpar-ent_list1	fu_fa_occ	5) What is currently your father’s occupation?	5) នាពេលបច្ចុប្បន្ននេះ តើឪពុករបស់ប្អូនប្រកបមុខរបរអ្វី?
select_one educ_list	fu_ma_att	6) What is the highest educational degree of your mother?	6) តើម្តាយរបស់ប្អូនរៀនបានថ្នាក់ទីប៉ុន្មាន?
select_multiple occpar-ent_list1	fu_ma_occ	7) What is currently your mother’s occupation?	7) នាពេលបច្ចុប្បន្ននេះ តើម្តាយរបស់ប្អូនប្រកបមុខរបរអ្វី?
select_one place_list	fu_fam_migr	8) Did any members of your close family work outside Cambodia in last twelve months?	8) តើមានសមាជិកក្រុមគ្រួសារសាច់ញាតិជិតស្និទ្ធិរបស់ប្អូនកំពុងធ្វើការងារនៅក្រៅប្រទេសកម្ពុជាដែរឬទេ ក្នុងកំឡុងពេល ១២ ខែនេះ?
note	fu_note_future	Now we would like to ask you a few questions about how you see yourself in the future...	ឥលូវនេះ បងសុំសួរពីរបីសំណួរទៅកាន់ប្អូន តើប្អូនចង់ឃើញអនាគតខ្លួនឯងដោយរបៀបណា..
select one yesmaybe_list	fu_ret_school	9) Are you considering to go back to school once they reopen?	9) តើប្អូនគិតថា ប្អូននឹងត្រឡប់ទៅរៀនវិញដែររឺទេ នៅពេលដែលសាលារៀនបើកដំណើរការដូចធម្មតា?
select_multiple dropout_list	fu_dropout_reason	9a) What is the main reason for you to have dropped out?	9a) តើបងអាចសុំសួរបានទេ ថាតើមូលហេតុអ្វីបានជាប្អូនត្រូវឈប់រៀន?
select_one aspeduc_list	fu_educ_asp	10) What is the highest level of formal education you would like to complete?	10) តើប្អូនៗចង់បញ្ចប់ការសិក្សា (អប់រំក្នុងប្រព័ន្ធ)នៅកម្រិតណា?
select_one aspeduc_list	fu_educ_asp_do	10a) If you could stay in school, what would be the highest level of formal education you would like to complete?	10a) ប្រសិនជាប្អូនមានឱកាសទៅសាលាវិញ តើប្អូនគិតថាប្អូនចង់បញ្ចប់ការសិក្សា (អប់រំក្នុងប្រព័ន្ធ)នៅកម្រិតណា?

select_one scale_list	fu_prob_educ	11) Given your and your family's situation, how likely do you think it is that you will achieve this level of education? Please indicate on this scale, where 0 is "Not likely at all" and 10 is "Very likely"	11) សូមប្រាប់យើងអំពីស្ថានភាពរបស់ប្អូន និងគ្រួសាររបស់ប្អូន ថាតើប្អូនគិតថាប្អូននឹងអាចសម្រេចបំណងប្រាថ្នាចំពោះការអប់រំនោះដល់កម្រិតណា?<br>សូមបញ្ជាក់នៅលើកម្រិតដែលយើងបានបង្ហាញនៅខាងក្រោម 0 គឺ «មិនអាចសម្រេចសេចក្តីប្រាថ្នាទាល់តែសោះ» និង 10 គឺ «ពិតជាអាចសម្រេចសេចក្តីប្រាថ្នានោះបាន»
select_one aspeduc_list	fu_educ_exp	12) Given your and your family's situation, what is the highest level of formal education you expect to complete?	12) សូមប្រាប់យើងអំពីស្ថានភាពរបស់ប្អូន និងគ្រួសាររបស់ប្អូន ថាតើការអប់រំ(ក្នុងប្រព័ន្ធ)ខ្ពស់បំផុតកម្រិតមួយណាដែលប្អូនរំពឹងថានឹងអាចសម្រេចទៅបាន?
select_one occkid_list	fu_pref_job	13) When you are about 25 years old, what job would you like to be doing?	13) នៅពេលដែលប្អូនមានអាយុ 25 ឆ្នាំ តើការងារអ្វីដែលប្អូនចង់ធ្វើ?
select_one scale_list	fu_prob_prefjob	14) Given your current situation, how likely do you think it is that you will be able to get that kind of job? Please indicate on this scale, where 0 is "Not likely at all" and 10 is "Very likely"	14) សូមប្រាប់យើងអំពីស្ថានភាពបច្ចុប្បន្នរបស់ប្អូន ថាតើប្អូនៗនឹងអាចទទួលបានការងារដែលប្អូនប្រាថ្នានោះយ៉ាងដូចម្តេច?<br>សូមបញ្ជាក់នៅលើកម្រិតដែលយើងបានបង្ហាញនៅខាងក្រោម 0 គឺ «មិនអាចសម្រេចសេចក្តីប្រាថ្នាទាល់តែសោះ» និង 10 គឺ «ពិតជាអាចសម្រេចសេចក្តីប្រាថ្នានោះបាន»
integer	fu_exp_hschool	15) How much would a student have to pay to go to high school per month? (incl. material, extra classes and transportation?	15) តើសិស្សម្នាក់ត្រូវចំណាយប្រាក់អស់ប៉ុន្មានក្នុងការបន្តការសិក្សានៅវិទ្យាល័យ សម្រាប់រយៈ ពេលមួយខែៗ (រួមបញ្ចូលទាំង សម្ភារៈសិក្សា រៀនគួរបន្ថែម និងការធ្វើដំណើរ)? សូមផ្តល់តម្លៃនៃការចំណាយជារូបិបណ្ណរៀល
select_one educcost_list	fu_share_costs	16) What is most expensive about going to high school?	16) តើអ្វីជាការចំណាយខ្ពស់បំផុតសម្រាប់ការសិស្សនៅថ្នាក់វិទ្យាល័យ?
calculate	fu_cost_label		
integer	fu_exp_mostimp	17) How much would a student have to pay for ${fu_cost_label} per month ?	17) ក្នុងមួយខែៗ តើសិស្សម្នាក់ចំណាយទៅលើ ${fu_cost_label} អស់ប៉ុន្មាន?
select_one scholship_list	fu_schol_applied	18) Did you apply for a high school scholarship?	18) តើប្អូនបានដាក់អាហាររូបករណ៍សម្រាប់ថ្នាក់វិទ្យាល័យដែរ រឺទេ?
note	fu_note_timeuse	Now we would like to ask you a few questions about what you did over the last weeks and months...	ឥលូវនេះ យើងសូមសួរពីរបីសំណួរ ថាតើអ្នកបានធ្វើអ្វីខ្លះកាលពីអាទិត្យមុន និងខែមុន....
select_one com_list	fu_contact_teach	19) When was the last time you heard from your teacher (in person, phone, facebook group, through class leader...)?	19) តើពេលណាជាពេលវេលាចុងក្រោយដែលប្អូនបានទាក់ទងជាមួយលោកគ្រូ/អ្នកគ្រូរបស់ប្អូន? (តាមផ្ទាល់ខ្លួន តាមមទូរស័ព្ទ ក្រុមហ្វេកបុក, តាមប្រធានថ្នាក់ ឬតាមប្រធានក្រុម)
select_one yes_list	fu_study_7days	20) Have you been engaged in education or learning activities over the last week?	20) កំឡុងពេល៧ថ្ងៃមុន តើប្អូនធ្លាប់បានរៀន ឬមានសកម្មភាពទាក់ទងនិងការរៀនសូត្រដែរឬទេ?

select_multiple educactivity_list	fu_study_type	21) In what types of education or learning activities have you been engaged in the last week?	21) កំឡុងពេល៧ថ្ងៃមុន តើសកម្មភាពអប់រំឬការរៀនសូត្របែបណាដែលប្អូនបានចូលរួម?
text	fu_study_type_other	Please specify the other types of learning activities...	សូមបញ្ជាក់អំពីប្រភេទនៃសកម្មភាពសិក្សាផ្សេងៗទៀត
select_one yes_list	fu_work_7days	22) Did you work in the last week?	22) កំឡុងពេល៧ថ្ងៃមុន តើប្អូនបានធ្វើការដែរ រឺទេ?
decimal	fu_hours_7days	23) On average how many hours per day?	23) ជាធម្យម តើប្អូនធ្វើការប៉ុន្មានម៉ោងក្នុងមួយថ្ងៃ?
select_one mainactivity_list	fu_mainact_7d	24) Think of a typical week-day (mo-fr) during last week. What was your main activity?	24) សូមគិតពីសប្តាហ៍មុន (ច័ន្ទ-សុក្រ) តើការងារអ្វីដែលជាការងារចំបងបំផុត ដែលប្អូនបានធ្វើ?
note	fu_note_covid1	In the following, we would like to learn about your experiences during the recent / current COVID-19 crisis (not only concerning the disease but the general situation in Cambodia)	ជាបន្ទាប់ យើងចង់ដឹងពីស្ថានភាពរបស់ប្អូន ក្នុងកំឡុងពេលថ្មីៗនេះ/ក្នុងកំឡុងពេលវិបត្តិ កូវីដ ១៩នេះ (មិនមែនសំដៅទៅលើតែការព្រួយបារម្ភអំពីជំងឺមួយមុខទេ ប៉ុន្តែអំពីស្ថានភាពទូទៅក្នុងប្រទេសកម្ពុជា)
select_one yes_list	fu_covid_kn	25) Have you heard about COVID-19/ Corona-virus?	25) តើប្អូនបានលឺ ឬបានដឹងអំពីកូវីដ ១៩ ឬ វីរុសកូរ៉ូណាដែររឺទេ?
note	fu_note_covid1_no	Covid-19 is the disease that caused the closing of the borders in March 2020 and the global economic crisis since.	វីរុសកូរ៉ូណា ឬកូវីដ ១៩ គឺជាមេរោគ ដែលបង្កឲ្យមានការបិទច្រកចេញចូលក្រៅប្រទេស តាំងពីខែមីនា ឆ្នាំ ២០២០ និងបានធ្វើឲ្យប៉ះពាល់យ៉ាងខ្លាំងដល់សេដ្ឋកិច្ចពិភពលោកទាំងមូល ។
select_one covidjob_list	fu_covid_incpar	26) Do one or both of your parents have lower income because of the COVID-19 crisis?	26) តើឪពុកម្តាយ ឬអាណាព្យាបាលរបស់ប្អូនទទួលបានចំណូលទាប ដោយសារតែវិបត្តិកូវីដ ១៩ នេះដែរ រឺទេ?
select_one covidjob_list	fu_covid_jobpar	27) Did one or both of your parents lose their job or change job because of the COVID-19 crisis?	27) តើក្នុងចំណោមឪពុកម្តាយ ឬអាណាព្យាបាលរបស់ប្អូន មាននរណាម្នាក់បានបាត់បង់ការងារ ឬបានផ្លាស់ប្តូរការងារដោយសារតែកូវីដ ១៩ ដែររឺទេ?
select_multiple occparent_list2	fu_covid_jobfa	28) What was your father doing before?	28) ពីមុន តើឪពុករបស់ប្អូនប្រកបមុខរបបអ្វី?
select_multiple occparent_list2	fu_covid_jobma	29) What was your mother doing before?	29) ពីមុន តើម្តាយរបស់ប្អូនប្រកបមុខរបបអ្វី?
select_multiple return_list	fu_covid_famret	30) You said earlier that someone from your close family worked outside Cambodia in the last 12 months. Were any of them forced to return because of the COVID-19 crisis?	30) ប្អូនបានប្រាប់បងថា សមាជិកក្រុមគ្រួសារសាច់ញាតិជិតស្និទ្ធិរបស់ប្អូនបានទៅធ្វើការងារនៅក្រៅប្រទេស ក្នុងរយៈពេល ១២ខែមុន តើមានអ្នកណាខ្លះត្រូវបានបង្ខំចិត្តត្រឡប់មកផ្ទះវិញដោយសារតែវិបត្តិ កូវីដ ១៩ ដែរ រឺទេ?

select_multiple covid-job_list2	fu_covid_changework	31) It is possible that you were working before the COVID-19 crisis, or not. Did that change due to the COVID-19 crisis?	31) វាអាច ដែលប្អូនមានការងារធ្វើមុនពេលវិបត្តិកូវីដ ១៩ ឬមិនមាន។ ដោយសារតែវិបត្តិកូវីដ ១៩ តើមានអ្វីផ្លាស់ប្តូរដែរ រឺទេ?
calculate	fu_educexp_label		
select_one educexp_list	fu_covid_expeduc	32) Remember that you said earlier that you expect to reach ${fu_educexp_label}. Has this changed due to the COVID-19 crisis?	32) មុននេះ ប្អូនបានប្រាប់អំពីការចង់បញ្ចប់ថ្នាក់ ${fu_educexp_label}. ដោយសារតែវិបត្តិកូវិដ ១៩ តើវាអាចធ្វើឲ្យមានការផ្លាស់ប្តូរដែររឺទេ?
note	fu_note_covid2	For the following statements with respect to the COVID-19 crisis, please indicate with the scale to which extent they apply for you.	សម្រាប់សំណួរខាងក្រោមទាក់ទងនឹងវិបត្តិ កូវីត១៩នេះ សូមបញ្ជាក់ពីទំហំដែលកូវីត១៩ប៉ះពាល់សម្រាប់អ្នក ។
select_one apply_list	fu_covid_finan	33) The COVID-19 crisis worsened the financial situation of my family	33) វិបត្តិកូវីដ ១៩ បានធ្វើឲ្យប៉ះពាល់យ៉ាងខ្លាំងដល់ស្ថានភាពហិរញ្ញវត្ថុគ្រួសាររបស់ប្អូន
select_one apply_list	fu_covid_reths	34) The COVID-19 crisis reduces the economic benefit of having a high school degree	34) វិបត្តិកូវីដ ១៩ បានកាត់បន្ថយលទ្ធភាពនៃការបន្តការសិស្សានៅថ្នាក់វិទ្យាល័យ
select_one apply_list	fu_covid_learn	35) During the COVID-19 school closure I keep studying for school	35) កំឡុងពេលនៃវិបត្តិកូវីដ ១៩ សាលាត្រូវបានបិទ តែខ្ញុំនៅតែបន្តការសិក្សា
select_one apply_list	fu_covid_oppco	36) There are no more well-paid jobs for lower secondary graduates (such as in in tourism or garment) due to the COVID-19 crisis	36) មិនមានការងារណា ដែលទទួលបានចំណូលខ្ពស់សម្រាប់សិស្សក្រោមថ្នាក់អនុវិទ្យាល័យទេ ព្រោះតែវិបត្តិកូវីដ ១៩ (ដូចជាការងារ នៅក្នុងផ្នែកទេសចរណ៍ ឬការងាររោងចក្រ)
select_one apply_list	fu_covid_health	37) The COVID-19 crisis increased my worry for my personal and my family's health	37) វិបត្តិកូវីដ ១៩ បានបង្កើនការព្រួយបារម្ភរបស់ខ្ញុំអំពីបញ្ហាសុខភាពរបស់ខ្ញុំ និងគ្រួសារខ្ញុំ ។
select_one apply_list	fu_covid_final	39) I am worried I will not be able to continue to grade 10 because of the COVID-19 crisis	39) ខ្ញុំបារម្ភថាខ្ញុំមិនអាចបន្តការសិក្សាទៅថ្នាក់ទី ១0បាន ដោយសារតែមេរោគកូវីដ ១៩
select_one apply_list	fu_covid_money	40) Because of the COVID-19 crisis, my family will not have the financial resources to allow me to go to high school	40) ដោយសារតែកូវីដ១៩ គ្រួសាររបស់ខ្ញុំនឹងមិនមានលទ្ធភាពគ្រប់គ្រាន់សម្រាប់ឲ្យខ្ញុំបន្តការសិក្សាទៅថ្នាក់វិទ្យាល័យបានទេ
select_one apply_list	fu_covid_hs	41) My motivation to go to high school increased due to COVID-19	41) ខ្ញុំមានការតាំងចិត្តនិងប្តេជ្ញាចិត្តទៅរៀនបន្តថ្នាក់វិទ្យាល័យ ច្រើនជាងមុន ដោយសារតែកូវីដ១៩
select_one apply_list	fu_covid_migr	42) Migrating for work is difficult due to the COVID-19 crisis	42) ការធ្វើចំណាកស្រុក ដើម្បីស្វែងរកការងារធ្វើមានការលំបាក ដោយសារតែកូវីដ ១៩
select_one apply_list	fu_covid_work	43) Because of the COVID-19 crisis, I had to start working in order to support my family financially	43) ដោយសារតែកូវីដ ១៩ ខ្ញុំត្រូវចាប់ផ្តើមធ្វើការ ដើម្បីជួយគាំទ្រ ហរិញ្ញវត្ថុគ្រួសាររបស់ខ្ញុំ

note	fu_note_end1	Thank you very much for your time and energy. This was very helpful!	សូមអរគុណដល់ប្អូនៗខ្លាំងណាស់ សម្រាប់ការចំណាយពេលវេលា និងថាមពលក្នុងការចូលរួមការស្ទង់មតិជាមួយយើង ។ វាពិតជាជួយយើងបានច្រើនណាស់ ។
note	fu_note_prize	As we had indicated at the beginning of our talk: From all students who participated in the survey 30 students will be randomly selected to receive a prize There are two prizes from which you can chose: (1) 15 US-$ phone credit that will be transferred directly to your phone. (2) Over-the-phone educational mentoring. In this mentoring program with experienced mentors from the NGO PEPY Empowering Youth, a personal mentor will help you in five one-hour sessions to develop your education related goals and show you strategies how you can achieve these goals. He will teach you best practices how you can study by yourself and manage your time most efficiently; and he will support you in detecting, solving and overcoming learning difficulties so you are very well prepared for school.<br>Out of all participating students, 30 students will be chosen randomly and will receive their chosen prize. The winners will be informed by phone, furthermore all winners will be announced on August 31 on the facebook page of PEPY empowering youth.	ដូចដែលបងបានប្រាប់ប្អូនពីដំបូងថា សិស្សទាំងអស់ដែលចូលរួមការស្ទង់មតិជាមួយយើង នឹងមានសិស្សចំនួន ៣០ នាក់ ដែលនឹងត្រូវបានឈ្នះរង្វាន់ពីការចូលរួមស្ទង់មតិ តាមរយៈការចាប់ឆ្នោត ។ រង្វាន់មាន 2 ប្រភេទ ដែលប្អូនអាចជ្រើសរើសបាន៖ 1. កាតទូរស័ព្ទដែលមានទឹកប្រាក់ចំនួន ១៥ ដុល្លារ ហើយនឹងត្រូវផ្ញើរទៅកាន់លេខទូរស័ព្ទរបស់ប្អូនដោយផ្ទាល់ 2. ការប្រឹក្សាយោបល់អំពីការសិក្សាតាមរយៈទូរស័ព្ទ នៅក្នុងកម្មវិធីនេះ ប្អូននឹងត្រូវបានប្រឹក្សាយោបល់ជាមួយលោកគ្រូអ្នកគ្រូដែលមានបទពិសោធន៍ធ្វើការងារជាច្រើនឆ្នាំ មកពីអង្គការលើកកម្ពស់យុវជន (ផេពភី) ។ អ្នកប្រឹក្សាជាយោបល់ និងធ្វើការជាមួយប្អូនចំនួន ៥ មេរៀន ដែលក្នុងមួយមេរៀនមានរយៈពេល ១ ម៉ោង ដើម្បីអាចឲ្យប្អូនអភិវឌ្ឍន៍ការសិក្សារបស់ប្អូនទាក់ទងនិងគោលដៅជីវិត និង បង្ហាញប្អូនអំពីវិធីសាស្រ្តក្នុងការសម្រេចគោលដៅ ។ ពួកគាត់និងបង្រៀនប្អូនអំពីវិធីសាស្រ្តដ៏ល្អ ដែលប្អូនអាចរៀនសូត្របានដោយខ្លួនឯងបាននៅផ្ទះ និងការគ្រប់គ្រង់ពេលវេលាដែលមានប្រសិទ្ធិភាពបំផុត ហើយពួកគាត់និងជួយគាំទ្រប្អូនក្នុងការដោះស្រាយបញ្ហា និងរបៀបនៃការជម្នះឧបសគ្គ ក្នុងការសិក្សារៀនសូត្ររបស់ប្អូនផងដែរ ដូច្នេះប្អូននិងមានការត្រៀមខ្លួនរួចរាល់សម្រាប់ការសិក្សារបស់ប្អូន ។ ក្នុងចំណោមអ្នកដែលបានចូលរួមជាមួយយើងទាំងអស់ និងមានសិស្សតែចំនួន ៣០ នាក់ប៉ុណ្ណោះ ដែលត្រូវទទួលបានរង្វាន់ ហើយនឹងត្រូវទទួលបានរង្វាន់ដែលខ្លួនបានជ្រើសរើសតែប៉ុណ្ណោះ ។ សម្រាប់អ្នកដែលបានឈ្នះរង្វាន់នេះ នឹងត្រូវបានផ្តល់ដំណឹងតាមរយៈការខល បន្ថែមពីនេះទៅទៀត យើងនិងធ្វើការប្រកាសតាមរយៈ គេហទំព័រហ្វេសបុករបស់អង្គការ លើកកម្ពស់យុវជន នៅថ្ងៃទី ៣០ ខែសីហា ឆ្នាំ ២០២០ ផងដែរ ។
select_one yes_list	fu_lottery	44) Would you like to be considered for these prizes?	44) តើប្អូនចង់រួមមួយជាមួយការចាប់រង្វាន់នេះដែររឺទេ?
select_one prize_list	fu_prize	45) Because the prize will be transferred automatically after winners are being chosen, you need to decide now which prize you would like to receive. Which one do you prefer?	45) ពីព្រោះរង្វាន់ និងត្រូវបានធ្វើការផ្តល់ជូនដោយស្វ័យប្រវិត្ត បន្ទាប់ពីការចាប់ឆ្នោតរួច ដូច្នេះប្អូនត្រូវសម្រេចចិត្តថា តើរង្វាន់មួយណាដែលប្អូនចង់បាន?

note	fu_note_contact	Thank you again for your participation. As the very last question, we would like to ask for your permission to talk to you again only one more time when schools reopened in Cambodia. This call will be realized by one of the survey team members. It will be very brief and is only necessary for a selected sample of students. If you do not wish to be contacted one more time in the future, this has no consequences for you and we will not reach out to you again.	អរគុណសម្រាប់ការចូលរួមក្នុងការស្ទង់មតិជាមួយបង ។ សុំសួរចុងក្រោយដែលបងចង់សួរប្អូន បងចង់សុំអនុញ្ញាតពីប្អូនក្នុងការទាក់ទងទៅកាន់ប្អូនម្តងទៀតនៅពេលដែលសាលារៀនបើកដំណើរការម្តងទៀត ។ ការទាក់ទងនេះនិងធ្វើឡើងដោយក្រុមរបស់ខាងបង វាជាការស្ទង់មតិខ្លីៗ ហើយតែសំណួរណាដែលសំខាន់ៗដែលត្រូវសួរ ហើយសម្រាប់ថាតែសិស្សមួយចំនួនតែប៉ុនណ្ណោះ ។ ប្រសិនជាប្អូនគិតថា ប្អូនមិនចង់ឲ្យបងទាក់ទងក៏បាន វាមិនប៉ះពាល់ដល់ប្អូន ហើយបងក៏មិនទាក់ទងទៀតដែ ។
select_one yes_list	fu_contact_next	Do you grant permission for my team to contact you one more time in the future?	ងអាចទាក់ទងទៅប្អូនម្តងទៀតបានទេ?
note	fu_note_end2	End of Interview	ការសម្ភាសន៍ត្រូវបានបញ្ចប់
note	fu_note_comments	After survey finished, to be filled out by interviewer.	បន្ទាប់ពីការស្ទង់មតិត្រូវបានបញ្ចប់ បំពេញដោយអ្នកសម្ភាសន៍
select_one apply_list	fu_assess_signal	46) The phone connection was very good (i.e. the call was not interrupted, it was not difficult to understand each other).	46) សេវារបស់ទូរស័ព្ទគឺល្អខ្លាំងណាស់ (ឧ. ការខលមិនមានការរំខាន មិនមានផលពិបាកក្នុងស្តាប់គ្នាទៅវិញទៅមក)
select_one apply_list	fu_assess_motiv	47) The student was very motivated.	47) សិស្ស គឺមានការតាំងចិត្តខ្លាំងណាស់
select_one apply_list	fu_assess_underst	48) The student understood the questions very well and had no difficulties in responding.	48) សិស្សយល់ច្បាស់ពីសំណួរ និងមិនមានភាពពិបាកក្នុងការឆ្លើយសំណួរ
text	fu_assess_comment	49) Any other comments	49) មតិយោបល់ផ្សេងៗ

Survey answers

list name	name	label::English (en)	label::Khmer (kh)
sex__list	1	male	ប្រុស
sex__list	2	female	ស្រី
sex__list	3	other	ផ្សេងៗ
sex__list	-77	Don’t know	មិនដឹង
sex__list	-88	Don’t want to answer	មិនចង់ឆ្លើយ
yes__list	1	yes	បាទ/ចាស
yes__list	2	no	ទេ
yes__list	-77	Don’t know	មិនដឹង
yes__list	-88	Don’t want to answer	មិនចង់ឆ្លើយ
yesmaybe__list	1	yes	បាទ/ចាស
yesmaybe__list	2	maybe	ប្រហែល/មិនច្បាស់
yesmaybe__list	3	no	ទេ
yesmaybe__list	-77	Don’t know	មិនដឹង
yesmaybe__list	-88	Don’t want to answer	មិនចង់ឆ្លើយ
educ__list	1	Illiterate / No education	មិនចេះអក្សរ/ មិនបានទទួលការអប់រំ
educ__list	2	Informal education (can read and write)	ការអប់រំក្រៅប្រព័ន្ធ (អាចអាន និងសរសេរបាន)
educ__list	3	Some primary (less than 6 years)	ថ្នាក់បឋមសិក្សា (តិចជាងថ្នាក់ទី ៦)
educ__list	4	Primary completed (6 years)	ថ្នាក់បឋមសិក្សា (បញ្ចប់ថ្នាក់ទី ៦)
educ__list	5	Lower secondary completed (9 years)	អនុវិទ្យាល័យ (បញ្ចប់ថ្នាក់ទី ៩)
educ__list	6	High school completed (12 years)	វិទ្យាល័យ (បញ្ចប់ថ្នាក់ទី ១២)
educ__list	7	University or higher (bachelor, master, phd)	បរិញ្ញាបត្រ ឬ អនុបណ្ឌិត ឬ បណ្ឌិត
educ__list	-77	Don’t know	មិនដឹង
educ__list	-88	Don’t want to answer	មិនចង់ឆ្លើយ
place__list	1	No	ទេ
place__list	2	Yes, in Thailand	បាទ/ចាស, នៅក្នុងប្រទេសថៃ
place__list	3	Yes, in other Countries	បាទ/ចាស, នៅប្រទេសផ្សេងទៀត
place__list	-77	Don’t know	មិនដឹង
place__list	-88	Don’t want to answer	មិនចង់ឆ្លើយ

aspeduc_list	1	Lower secondary	អនុវិទ្យាល័យ
aspeduc_list	2	Higher secondary	វិទ្យាល័យ
aspeduc_list	3	Vocational training, such as mechatronics technician, hair-dresser	មជ្ឈមណ្ឌលបណ្តុះបណ្តាលជំនាញ និងវិជ្ជាជីវៈ(ជាងជុសជុលអគ្គសនី, ជាងកាត់សក់,...)
aspeduc_list	4	University, Bachelor's	សាកលវិទ្យាល័យ បរិញ្ញាបត្រ
aspeduc_list	5	University, Master's	សាកលវិទ្យាល័យ អនុបណ្ឌិត
aspeduc_list	6	University, PhD	សាកលវិទ្យាល័យ បណ្ឌិត
aspeduc_list	-77	Don't know	មិនដឹង
aspeduc_list	-88	Don't want to answer	មិនចង់ឆ្លើយ
dropout_list	1	Performance in school not good enough to continue	ពិន្ទុនៅសាលាមិនសូវល្អ
dropout_list	2	Family cannot afford it	គ្រួសារមិនអាចផ្គត់ផ្គង់ការរៀនបន្ត
dropout_list	3	Want to earn money	ចង់រកលុយ
dropout_list	4	Need to earn money for family	ត្រូវរកលុយជួយគ្រួសារ
dropout_list	5	No value in lower secondary-level education/ grade 9 diploma	មើលមិនឃើញភាពសំខាន់/អត្ថប្រយោជន៍នៃការសិក្សាថ្នាក់អនុវិទ្យាល័យ
dropout_list	50	OTHER (SPECIFY)	ផ្សេងៗ (ចូរបញ្ជាក់)
dropout_list	-77	Don't know	មិនដឹង
dropout_list	-88	Don't want to answer	មិនចង់ឆ្លើយ
educcost_list	1	transportation	ការធ្វើដំណើរ
educcost_list	2	accommodation and food	ការស្នាក់នៅ និងម្ហូបអាហារ
educcost_list	3	extra classes	ថ្នាក់រៀនបន្ថែម
educcost_list	4	school material	សម្ភារៈសិក្សា
educcost_list	-77	Don't know	មិនដឹង
educcost_list	-88	Don't want to answer	មិនចង់ឆ្លើយ
scholship_list	1	Yes	បាទ/ចាស
scholship_list	2	No, no sholarship was available this year	ទេ មិនមានអាហារូបករណ៍ទេសម្រាប់ឆ្នាំនេះ
scholship_list	3	No, did not know of any scholarships	ទេ មិនស្គាល់អាហារូបករណ៍ណាមួយទេ
scholship_list	4	Don't know what a scholarship is	មិនស្គាល់អ្វីទៅជាអាហារូបករណ៍
scholship_list	5	No, for other reasons	ទេ ដោយសារមូលផ្សេង
scholship_list	-88	Don't want to answer	មិនចង់ឆ្លើយ
com_list	1	Within the last week	កំឡុងពេល៧ថ្ងៃមុន
com_list	2	Within the last month	កំឡុងពេលមួយខែមុន

com_list	3	More than a month ago	ជាងមួយខែមុន
com_list	4	Before schools were closed	មុនពេលសាលាបិទ
com_list	-77	Don’t know	មិនដឹង
com_list	-88	Don’t want to answer	មិនចង់ឆ្លើយ
educactivity_list	1	Completed assignments provided by the teacher (telegram, facebook)	ធ្វើកិច្ចការផ្ទះ ដែលលោកគ្រូ/អ្នកគ្រូបានផ្តល់ឱ្យ (តាម Telegram, facebook)
educactivity_list	2	Watched educational TV programs	មើលកម្មវិធីមេរៀនតាមទូរទស្សន៍
educactivity_list	3	Study with textbooks/ material (no specific assignment)	រៀនតាមសៀវភៅពុម្ព/ឯកសារផ្សេងៗ (មិនមានកិច្ចការសាលាច្បាស់លាស់)
educactivity_list	4	Study in small group	រៀនតាមក្រុមតូចៗ
educactivity_list	5	In-person session/meeting with teacher	ជួបជុំរៀនមេរៀន ជាមួយគ្រូសាលារដ្ឋ
educactivity_list	6	Online session/meeting with teacher	រៀនតាមអេនឡាញ ជាមួយគ្រូសាលារដ្ឋ
educactivity_list	7	Session/ meeting with tutor	មេរៀន/ការជួបជាមួយគ្រូបង្រៀនបង់ថ្លៃ
educactivity_list	50	OTHER (SPECIFY)	ផ្សេងៗ(ចូរបញ្ជាក់)
educactivity_list	-77	Don’t know	មិនដឹង
educactivity_list	-88	Don’t want to answer	មិនចង់ឆ្លើយ
mainactivity_list	1	study	រៀន
mainactivity_list	2	work for pay	ធ្វើការងារដើម្បីរកចំណូល
mainactivity_list	3	work on family farm/business	ការងារធ្វើស្រែ ឬលក់ដូរ ក្នុងគ្រួសារ
mainactivity_list	4	do household chores (incl. caring for younger siblings)	ធ្វើការងារផ្ទះ (មើលប្អូន ...)
mainactivity_list	5	ran any kind of business	រកស៊ី ឬលក់ដូរ
mainactivity_list	6	fishing, collecting firewook	រកត្រី រកអុស
mainactivity_list	7	meet with friends or go to the pagoda	ជួបជុំមិត្តភ័ក្ក ឬក៏ទៅវត្ត
mainactivity_list	8	leisure (s.a. play on the phone or outside)	ដើរលេងកំសាន្ត (លេងទូរស័ព្ទ.....ដើរលេងខាងក្រៅផ្សេងៗ)
mainactivity_list	50	OTHER (SPECIFY)	ផ្សេងៗ(ចូរបញ្ជាក់)
mainactivity_list	-77	Don’t know	មិនដឹង
mainactivity_list	-88	Don’t want to answer	មិនចង់ឆ្លើយ
covidjob_list	1	Yes – both	បាទ/ចាស (ទាំងពីរនាក់)
covidjob_list	2	Yes – mother only	បាទ/ចាស (តែម្តាយ)
covidjob_list	3	Yes – father only	បាទ/ចាស (តែឪពុក)
covidjob_list	4	No	ទេ
covidjob_list	-77	Don’t know	មិនដឹង
covidjob_list	-88	Don’t want to answer	មិនចង់ឆ្លើយ
return_list	1	No	ទេ

return_list	2	Yes – father	បាទ/ចាស (ម្ដាយ)
return_list	3	Yes- mother	បាទ/ចាស (ឪពុក)
return_list	4	Yes – other close family members	បាទ/ចាស សាច់ញាតិជិតស្និទ្ធ
return_list	-77	Don't know	មិនដឹង
return_list	-88	Don't want to answer	មិនចង់ឆ្លើយ
covidjob_list2	1	Yes – lost job	បាទ/ចាស បាត់បង់ការងារ
covidjob_list2	2	Yes – work less hours	បាទ/ចាស មានការងារម៉ោងតិចជាងមុន
covidjob_list2	3	Yes – started new job	បាទ/ចាស បានចាប់ផ្ដើមការងារថ្មី
covidjob_list2	4	Yes – work more hours	បាទ/ចាស ធ្វើការងារច្រើនម៉ោងជាងមុន
covidjob_list2	5	No	ទេ
covidjob_list2	-77	Don't know	មិនដឹង
covidjob_list2	-88	Don't want to answer	មិនចង់ឆ្លើយ
educexp_list	1	yes, became higher	បាទ/ចាស ខ្ពស់ជាងមុន
educexp_list	2	yes, became lower	បាទ/ចាស ទាបជាងមុន
educexp_list	3	no, about the same	ទេ នៅធម្មតា
educexp_list	-77	Don't know	មិនដឹង
educexp_list	-88	Don't want to answer	មិនចង់ឆ្លើយ
apply_list	1	Applies very much	ត្រូវខ្លាំង
apply_list	2	Applies a little	ត្រូវមធ្យម
apply_list	3	Does not apply very much	ត្រូវបន្តិចបន្តួច
apply_list	4	Does not apply at all	មិនត្រូវទាល់តែសោះ
apply_list	-77	Don't know	មិនដឹង
apply_list	-88	Don't want to answer	មិនចង់ឆ្លើយ
scale_list	0	0	0
scale_list	1	1	1
scale_list	2	2	2
scale_list	3	3	3
scale_list	4	4	4
scale_list	5	5	5
scale_list	6	6	6
scale_list	7	7	7
scale_list	8	8	8
scale_list	9	9	9

scale_list	10	10	10
scale_list	-77	Don’t know	មិនដឹង
scale_list	-88	Don’t want to answer	មិនចង់ឆ្លើយ
occparent_list1	1	Stay at home	នៅផ្ទះ
occparent_list1	2	Farmer (crop, livestock)	កសិករ
occparent_list1	3	Work in Forestry	ធ្វើការក្នុងផ្នែកព្រៃឈើ
occparent_list1	4	Fishery worker	អ្នកនេសាទ
occparent_list1	5	Agricultural laborer (incl. Fruit pickers etc)	បុគ្គលិកផ្នែកកសិកម្ម (រួមមានអ្នកបេះផ្លែឈើ...... ។ល។)
occparent_list1	6	Handcraft (weaving, basket maker, sculpturing...)	សិប្បកម្ម (អ្នកតម្បាញ, អ្នកធ្វើកន្ត្រក,ជាងចម្លាក់....)
occparent_list1	7	Own business (shop owner, street vendor)	ម្ចាស់អាជីវកម្ម
occparent_list1	8	Food processing (chef, baker, etc.)	អ្នកកែច្នៃចំណីអាហារ (ចុងភៅ អ្នកដុតនំ.... ។ល។)
occparent_list1	9	Work in tourism (tour guide etc)	ធ្វើការក្នុងផ្នែកទេសចរណ៍ (មគ្គុទេសក៍ទេសចរណ៍... ។ល។)
occparent_list1	10	Driver (tuktuk, taxi)	អ្នកបើកបរ (អ្នករត់តុកតុក,អ្នកបើកឡាន)
occparent_list1	11	Construction worker	កម្មករសំណង់
occparent_list1	12	Garment factory worker	កម្មកររោងចក្រ
occparent_list1	13	Mining worker	កម្មកររ៉ែ
occparent_list1	14	Office clerk	បុគ្គលិកការិយាល័យ
occparent_list1	15	Government employee	មន្ត្រីរាជការ
occparent_list1	16	Teacher	គ្រូបង្រៀន
occparent_list1	17	Police officer	មន្ត្រីប៉ូលីស
occparent_list1	18	Military/ Soldier	យោធា/ទាហាន
occparent_list1	40	Deceased	ស្លាប់
occparent_list1	50	OTHER (SPECIFY)	ផ្សេងៗ(ចូរបញ្ជាក់)
occparent_list1	-77	Don’t know	មិនដឹង
occparent_list1	-88	Don’t want to answer	មិនចង់ឆ្លើយ
occkid_list	1	Primary-level Teacher	គ្រូកម្រិតបឋមសិក្សា
occkid_list	2	Lower-secondary teacher	គ្រូកម្រិតអនុវិទ្យាល័យ
occkid_list	3	High school teacher	គ្រូកម្រិតវិទ្យាល័យ
occkid_list	4	Police officer	មន្ត្រីប៉ូលីស
occkid_list	5	Military/ Soldier	យោធា/ទាហាន
occkid_list	6	NGO staff/ social worker	អ្នកបំរើការងារសង្គម
occkid_list	7	Lawyer	មេធាវី
occkid_list	8	General practitioner	គ្រូពេទ្យបង្អែក
occkid_list	9	Nurse	គិលានុបដ្ឋាយិកា
occkid_list	10	Agricultural technician	អ្នកបច្ចេកទេសកសិកម្ម
occkid_list	11	Civil engineer	វិស្វករ
occkid_list	12	Electrician	ជាងអគ្គិសនី

occkid_list	13	Motorbike repairer	ជាងម៉ូតូ
occkid_list	14	Tour guide	មគ្គុទេសក៍ទេសចរណ៍
occkid_list	15	Photographer	អ្នកថតរូប
occkid_list	16	Make-up artist	អ្នកផាត់មុខ
occkid_list	17	Clothes designer/Tailor	អ្នកច្នៃម៉ូត/ជាងកាត់ដេរ
occkid_list	18	Artist/ Singer	សិល្បករ/អ្នកចម្រៀង
occkid_list	19	Chef	មេចុងភៅ
occkid_list	20	Banker	បុគ្គលិកធានាគារ
occkid_list	21	Office Administrator	អ្នកគ្រប់គ្រងការិយាល័យ
occkid_list	22	Software developer	អ្នកបង្កើតកម្មវិធីកុំព្យូទ័រ
occkid_list	23	Business owner	ម្ចាស់អាជីវកម្ម
occkid_list	50	OTHER (SPECIFY)	ផ្សេងៗ(ចូរបញ្ជាក់)
occkid_list	-77	Don't know	មិនដឹង
occkid_list	-88	Don't want to answer	មិនចង់ឆ្លើយ
occparent_list2	1	Stay at home	នៅផ្ទះ
occparent_list2	2	Farmer (crop, livestock)	កសិករ
occparent_list2	3	Work in Forestry	ធ្វើការក្នុងផ្នែកព្រៃឈើ
occparent_list2	4	Fishery worker	អ្នកនេសាទ
occparent_list2	5	Agricultural laborer (incl. Fruit pickers etc)	បុគ្គលិកផ្នែកកសិកម្ម (រួមមានអ្នកបេះផ្លែឈើ...... ។ល។)
occparent_list2	6	Handcraft (weaving, basket maker, sculpturing...)	សិប្បកម្ម (អ្នកតម្បាញ, អ្នកធ្វើកន្ត្រក,ជាងចម្លាក់....)
occparent_list2	7	Own business (shop owner, street vendor)	ម្ចាស់អាជីវកម្ម
occparent_list2	8	Food processing (chef, baker, etc.)	អ្នកកែច្នៃចំណីអាហារ (ចុងភៅ អ្នកដុតនំ.... ។ល។)
occparent_list2	9	Work in tourism (tour guide etc)	ធ្វើការក្នុងផ្នែកទេសចរណ៍ (មគ្គុទេសក៍ទេសចរណ៍... ។ល។)
occparent_list2	10	Driver (tuktuk, taxi)	អ្នកបើកបរ (អ្នករត់តុកតុក,អ្នកបើកឡាន)
occparent_list2	11	Construction worker	កម្មករសំណង់
occparent_list2	12	Garment factory worker	កម្មកររោងចក្រ
occparent_list2	13	Mining worker	កម្មកររ៉ែ
occparent_list2	14	Office clerk	បុគ្គលិកការិយាល័យ
occparent_list2	15	Government employee	មន្ត្រីរាជការ
occparent_list2	16	Teacher	គ្រូបង្រៀន
occparent_list2	17	Police officer	មន្ត្រីប៉ូលីស
occparent_list2	18	Military/ Soldier	យោធា/ទាហាន
occparent_list2	50	OTHER (SPECIFY)	ផ្សេងៗ(ចូរបញ្ជាក់)
occparent_list2	-77	Don't know	មិនដឹង
occparent_list2	-88	Don't want to answer	មិនចង់ឆ្លើយ
prize_list	1	Phone credit	កាតទូរស័ព្ទ

prize_list	2	Educational mentoring	ការប្រឹក្សាយោបល់អំពីការសិក្សា
